# Functional model for amelogenesis: polarization and pH sensitivity of calcium uptake in ameloblast-derived HAT-7 cells

**DOI:** 10.1007/s00223-026-01559-x

**Published:** 2026-06-10

**Authors:** Kristóf Kádár, Anna Földes, Róbert Rácz, Susan Van-Weert, Ádám Soós, Jason Bruce, Martin C. Steward, Pamela DenBesten, Gábor Varga, Ákos Zsembery

**Affiliations:** 1 Department of Oral Biology, Semmelweis University, Nagyvárad tér 4, Budapest, 1089 Hungary; 2 Department of Anatomy, Histology and Embryology, Semmelweis University, Budapest, Hungary; 3 School of Medical Sciences, Faculty of Biology, Medicine and Health, University of Manchester, Manchester, UK; 4 Department of Orofacial Science, University of California, San Francisco, USA

**Keywords:** Amelogenesis, Epithelial polarity, Ca^2+^ transport, SOCE, TRPM7, Extracellular pH

## Abstract

Enamel mineralization requires calcium (Ca^2+^) entering the matrix through the epithelium. The exact transepithelial Ca^2+^ transport process remains unclear. We have previously shown that rat-derived HAT-7 ameloblast cells, cultured in a polarized condition, achieve transepithelial bicarbonate transport. Our current aim was to determine the functional polarization of known amelogenesis-related Ca^2+^ transporters and channels in polarized HAT-7 cells. HAT-7 cells were cultured on Transwell membranes. The formation of a confluent monolayer was monitored by measuring transepithelial resistance. Relative mRNA expression of calcium channels was compared using TaqMan qPCR assays. Intracellular Ca^2+^ responses were followed by ratiometric Ca^2+^-imaging (fura-2 AM). Store-operated Ca^2+^ entry (SOCE) was activated by store depletion, using the SERCA inhibitor thapsigargin, while TRPM7 function was evaluated using the agonists naltriben and mibefradil. Functional polarization was determined by separate perfusion of the apical and basolateral membrane domains. The effects of both apical and basolateral extracellular acidification on calcium transport were also assessed. HAT-7 cells exhibited high Trpm7 mRNA expression, although much of the TRPM7 protein was localised to intracellular organelles. mRNAs for the SOCE channels Orai1, Orai2 and Orai3 were also detected. Both SOCE and TRPM7 activity showed functional polarization: SOCE being more prominent on the basolateral side while the TRPM7 activity was predominantly observed apically. Basolateral but not apical extracellular acidification reduced the basolateral SOCE-related Ca^2+^ entry. In summary, Ca^2+^ uptake mechanisms showed significant functional polarization in confluent HAT-7 monolayers, suggesting that HAT-7 cells may provide a model for transepithelial Ca^2+^ transport during enamel maturation.

**Supplementary Information:**

The online version contains supplementary material available at 10.1007/s00223-026-01559-x.

## Introduction

Ameloblasts are specialized epithelial cells that produce tooth enamel, a heavily mineralized tissue characterized by a highly ordered crystalline structure. To achieve this, calcium is transported across the ameloblast layer from the blood to the enamel matrix. Because the concentration of Ca^2+^ in the cytosol is held at an extremely low level, Ca^2+^ secretion must require active Ca^2+^ extrusion at the apical membrane and a corresponding, likely passive, Ca^2+^ uptake at the basolateral membrane. Known extrusion mechanisms include plasma membrane Ca^2+^-ATPases (PMCAs) and Na^+^/Ca^2+^ exchangers (NCX and NCKX families) [1], with different transporters dominating at different stages of amelogenesis [2].

Calcium uptake in ameloblasts is largely attributed to store-operated Ca^2+^ entry (SOCE) [3, 4], a major Ca^2+^ influx mechanism in both excitable and non-excitable cells. Additional pathways, such as uptake through the divalent cation-permeable TRPM7 channel, have also been suggested [5]. TRPM7 shows high mRNA expression in teeth [6], and deletion of its kinase domain leads to hypomineralized enamel with reduced volume [7]. Some studies suggest that TRPM7 may not act as a direct Ca^2+^ entry channel in ameloblasts but instead modulates Ca^2+^ entry via SOCE [8, 9]. However, its pH and Mg^2+^ sensitivity [10] support a potential regulatory role in amelogenesis. Because ameloblast maturation and the final mineralization of the enamel are accompanied by characteristic cycles of acidification in the enamel matrix [11], Ca^2+^ transport and pH regulation are likely to be tightly interconnected in these cells.

Maintaining a stable cytosolic Ca^2+^ concentration during transcellular Ca^2+^ transport requires the coordinated action of Ca^2+^ uptake and extrusion mechanisms at the basolateral and apical membranes respectively. However, due to the lack of robust in vitro models—particularly the need for polarized ameloblast monolayers suitable for physiological measurements—our understanding of the interplay between transporters and channels during vectorial Ca^2+^ transport in amelogenesis remains limited.

Therefore, the present study aimed to functionally characterize and determine the membrane polarization of Ca^2+^ uptake pathways in polarized HAT-7 ameloblast-derived cells originated from the cervical loop of rat incisor [12]. When cultured on permeable Transwell polyester membranes, these cells form polarized, confluent monolayers and retain key ameloblast properties. They establish functional tight junctions, as shown by their high transepithelial electrical resistance (TER) and the expression of claudin-1, -4, and -8 tight junction proteins. HAT-7 cells also express marker proteins characteristic of maturation-stage ameloblasts. Importantly, mounting HAT-7 monolayers between separately perfused apical and basal chambers enables the demonstration of vectorial basolateral-to-apical bicarbonate transport and the localization of the specific transporters involved in this process [13]. Specifically, we investigated whether distinct Ca^2+^ entry mechanisms display preferential localization to either the apical or the basolateral membrane, thereby contributing to vectorial Ca^2+^ transport during enamel maturation. We provide evidence that HAT-7 cells grown as polarized monolayers show regulated Ca^2+^ uptake through both SOCE and TRPM7 channels but at different cellular locations.

## Methods

### Cell culture

HAT-7 cells [12] were maintained in Dulbecco’s modified Eagle’s medium with Nutrient Mixture F-12 (DMEM: F12; Sigma Aldrich) supplemented with 10% foetal bovine serum (FBS; HyClone, Gibco), 100 U/ml penicillin, and 100 µg/ml streptomycin (Sigma Aldrich) under standard culture conditions (37 °C, 5% CO_2_). Cells were subcultured at regular intervals using 0.25% trypsin-EDTA (Gibco) then seeded onto permeable polyester Transwell inserts (0.4 μm pore size, 12 mm diameter; Transwell^®^/Snapwell™, Corning) and cultured for 2 days in standard medium followed by 3 days in differentiation medium (standard medium supplemented with 2.1 mM CaCl_2_ and 10^−5^ mM dexamethasone; Sigma). Cells between passages 5 and 25 were used for the experiments. Transepithelial electrical resistance (TER) was measured daily using an epithelial volt-ohm meter (EVOM, World Precision Instruments) to assess barrier function and paracellular electrolyte permeability.

### RT-qPCR

For mRNA expression studies, cells were cultured on Transwell membranes for either 5 days in standard medium (“polarized”) or for 2 days in standard medium followed by 3 days in differentiation medium (“diff polarized”). Alternatively, cells were harvested directly from plastic culture dishes (“unpolarized”). Total RNA was extracted using the GeneJET RNA Purification Kit (Thermo Fisher Scientific). RNA integrity was verified on 1% agarose gels, and cDNA was synthesized using a Maxima First Strand cDNA Synthesis Kit (Thermo Fisher Scientific). qPCR was performed on an ABI StepOne or QuantStudio 5 system using either TaqMan Universal Master Mix II or TaqPath qPCR Master Mix, CG (Applied Biosystems). The following TaqMan assays were used (Life Technologies Magyarország Kft): Trpm7 (Rn01328216m1), Orai1-3 (Rn02397170_m1, Rn01480748_m1, Rn01774170_m1). The acidic ribosomal protein P0 (RPLP0; Rn00821065_g1) was used as an internal reference and each sample was measured in technical triplicates. To calculate relative fold changes the comparative Ct method (2^−ΔΔCT^) was used.

### Immunocytochemistry

**Fig. 1:**
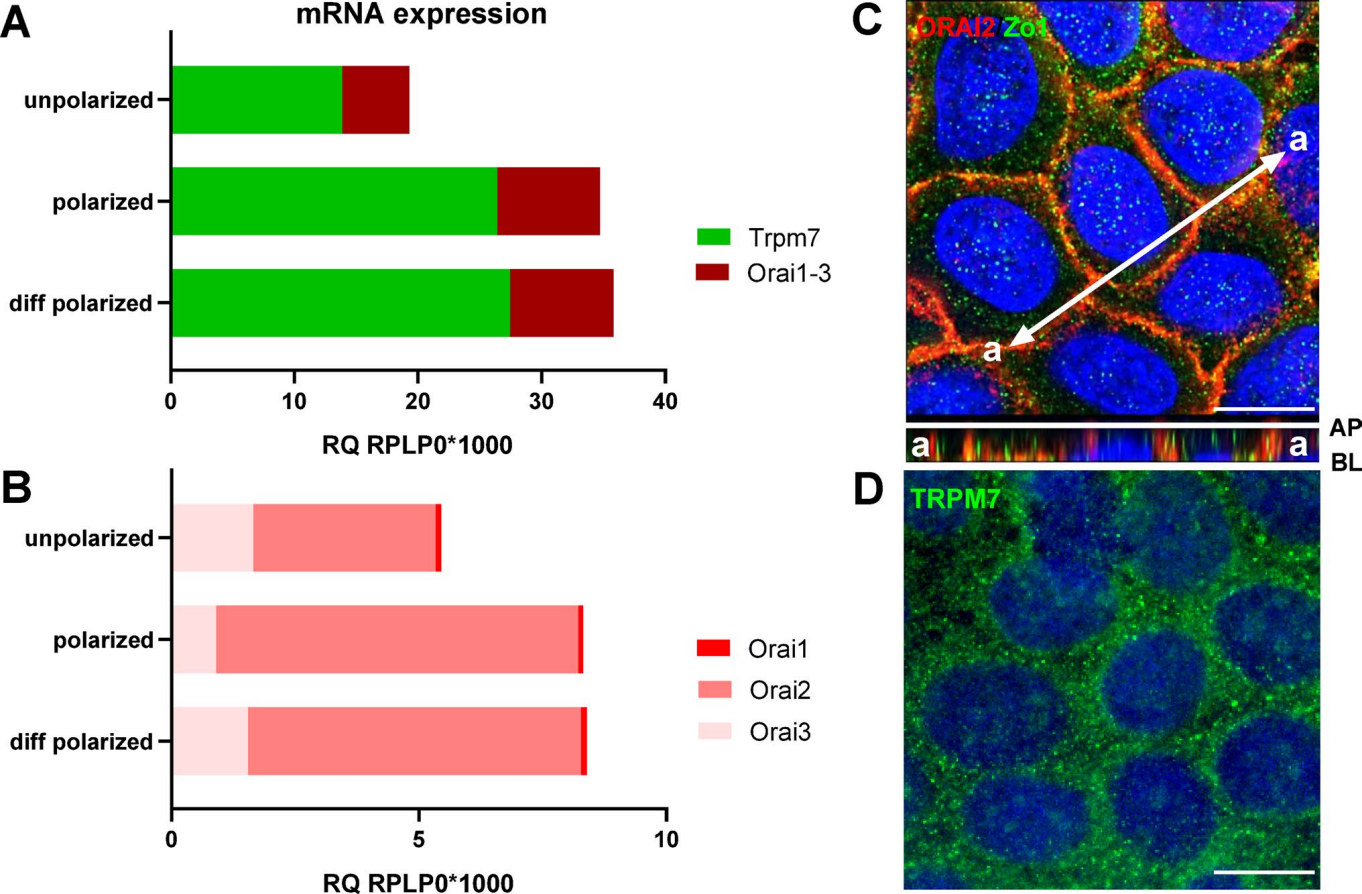
Expression pattern of Trpm7 and Orai1-3 in HAT-7 cells. **A** mRNA expression of Trpm7 and total Orai1-3 in cells cultured on plastic (unpolarized) or on Transwell membranes (polarized) with standard or differentiating (diff) medium, normalized to housekeeping gene RPLP0. **B** Expression of individual Orai isoforms normalized to housekeeping gene RPLP0 (**C**–**D**) Immunolocalization of Ca^2+^ channel proteins in polarized HAT-7 cells grown on Transwell membranes with differentiating medium. **C** ORAI2 (red) and tight junction protein ZO-1 (green) in a 3D confocal z-stack; insert shows a cross-section along line a–a (AP: apical, BL: basal). **D** TRPM7 protein (green). Nuclei counterstained with DAPI (blue) and scale bar 10 μm in both panels, for staining controls see Supplementary Fig. 1

HAT-7 cells grown on Transwell membranes were fixed with 4% paraformaldehyde at room temperature (RT) for 20 min. Permeabilization was achieved with 0.1% Triton X-100 applied for 10 min, and non-specific binding was blocked with 5% donkey serum at RT for 1 h. The membranes were incubated overnight at 4 °C with rabbit anti-TRPM7 (Thermo Fisher Scientific, ACC-047; 1:100), followed by a 1 h incubation at RT with Alexa Fluor 488-labeled donkey anti-rabbit IgG (Invitrogen; 1:250). In colocalization studies membranes were incubated overnight at 4 °C with rabbit anti-ZO-1 (Invitrogen, 40-2200, 3ug/ml) followed by a 1 h incubation at RT with Alexa Fluor 488-labeled donkey anti-rabbit IgG (Invitrogen; 1:250). They were then incubated overnight at 4 °C with mouse monoclonal ORAI2 (G-5) antibody (Santa Cruz, sc-376757, 1:100) followed by a 1 h incubation at RT with Alexa Fluor 594-labelled donkey anti-mouse IgG (Invitrogen; 1:250). Negative controls were prepared by omitting the primary antibody during the first incubation step. Nuclei were counterstained with DAPI (1 µg/ml working concentration; Merck) for 25 min. The HAT-7 monolayers were imaged by confocal scanning (Zeiss LSM 900) and stimulated emission depletion (STED, Abberior Expert Line- Nikon Ti2) microscopy using CellSens (Evident/Olympus) and ZEN Imaging (Zeiss) software. Image processing was performed in ImageJ and Adobe Photoshop CC 2020 (Adobe). Positive control stainings were performed on appropriate rat tissue cryosections (see Supplemetary Fig. 1).

### Ca^2+^ imaging

HAT-7 cells grown on Snapwell™ inserts (Corning) were loaded with 4 µM fura-2 AM (Invitrogen), 0.08% F-127 (Sigma), and 1 mM probenecid (Molecular Probes) in standard bath solution (137 mM NaCl, 5 mM KCl, 2 mM CaCl₂, 1 mM MgCl₂, 10 mM HEPES, and 10 mM glucose, adjusted to pH 7.4 with NaOH) for 75–80 min at 37 °C, followed by 20–30 min in fura-2-free, probenecid-containing bath solution to allow dye de-esterification. These solutions were applied to both apical and basolateral compartments, with probenecid included to prevent fura-2 efflux. Inserts were transferred to a custom imaging chamber enabling separate perfusion of the apical and basolateral sides and mounted on an upright fluorescence microscope, which minimizes the background signal from the autofluorescence of the insert membrane. Cells were excited alternately at 340 and 380 nm through a 20× water-immersion objective (NA 0.5, Nikon) using a metal-halide lamp with internal filter wheel (Prior Lumen 220 Pro). Images were acquired with an sCMOS camera (Prime BSI, Teledyne Photometrics) controlled by NIS AR software (Nikon). Intracellular Ca^2+^ changes were expressed as F340/F380 ratios normalized to baseline, with quantification performed over ROIs containing at least 50 cells. The Ca^2+^ influx rate was estimated by calculating the maximum slope of the rising phase of the curve from its first derivative. Briefly, the time-series curve was differentiated, and the three highest consecutive values of the first derivative were averaged to define the peak influx rate.

### Intracellular pH measurements

Real-time intracellular pH (pH_i_) monitoring was carried out by microfluorometry using the pH-sensitive dye BCECF as described previously [13]. HAT-7 cells grown on Transwell inserts were loaded with 4 µM BCECF AM (Thermo Fisher Scientific) for 30 min, then mounted in a perfusion chamber on a Nikon Eclipse TE200 inverted fluorescence microscope. Cells were superfused on both sides at 3 ml/min with the same bath solution as that used for Ca^2+^ imaging. Fluorescence was excited alternately at 490 nm (pH-sensitive) and 440 nm (pH-insensitive), and the emission recorded at 530 nm with a photomultiplier/amplifier system (Cairn Research). Data were acquired with DASYLab software (Measurement Computing) and F490/F440 fluorescence ratios were calculated every 5 s. Autofluorescence was corrected at the end of each experiment by permeabilizing cells with Triton X-100, and calibration was performed using the high K⁺/nigericin technique [14].

### Statistical analysis

Data are presented as the mean ± SEM. Statistical analyses included planned pairwise comparisons (using paired or unpaired two-sample t-tests), one-sample t-tests and ANOVA with Dunnett’s post hoc test. Differences were considered significant at *p* < 0.05.

## Results

### Both TRPM7 and ORAI channels are abundantly expressed in polarized HAT-7 cells

Quantitative gene expression analysis showed strong expression of Trpm7 and Orai1-3 mRNAs in unpolarized HAT-7 cells grown on plastic, with Trpm7 the most abundant. Polarized HAT-7 cell monolayers grown on permeable Transwell membranes, in both standard and differentiation medium, showed markedly upregulated Trpm7 and moderately increased total Orai channel expression (Fig. 1A). Comparing the different Orai isoforms, we found that Orai2 is the most abundant while Orai1 exhibits the lowest levels of expression with an Orai2/Orai1 ratio of 54.2 and Orai3/Orai1 ratio of 12.5 in polarized cells grown in differentiation medium (Fig. 1B). Immunocytochemistry revealed prominent membrane localization of ORAI2 in HAT-7 monolayers, with ZO-1 co-labelling of the tight junctions indicating a predominantly lateral membrane distribution of ORAI2 (Fig. 1C and insert). In contrast, under the same polarizing conditions, TRPM7 protein displayed a diffuse, largely intracellular expression (Fig. 1D). Functional studies were performed on polarized cells grown in differentiation media.

### Store-operated Ca^2+^ entry and TRPM7-mediated Ca^2+^ uptake show functional polarization in HAT-7 monolayers

**Fig. 2:**
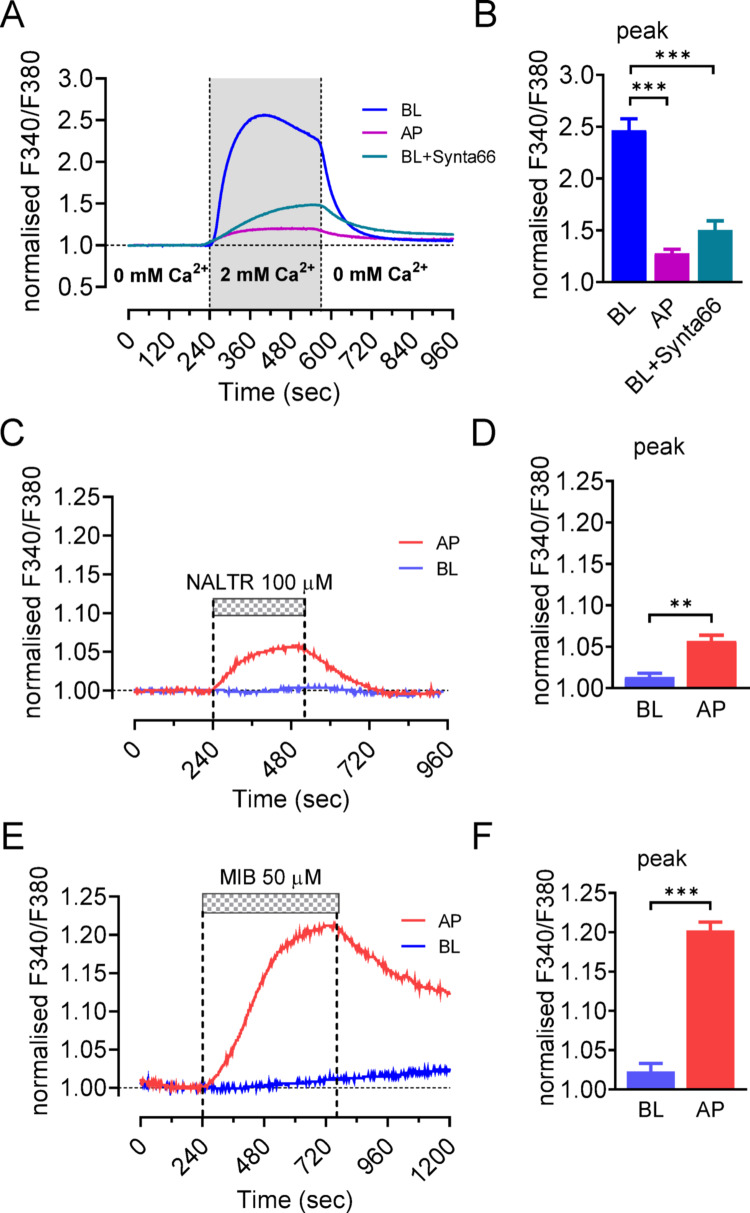
Intracellular Ca^2+^ measurements in HAT-7 cell monolayers cultured on Transwell membranes. **A**-**B** Intracellular Ca^2+^ changes elicited by basolateral (BL, *n* = 7) or apical (AP, *n* = 5) restoration of extracellular Ca^2+^ in ER store-depleted HAT-7 cell monolayers following thapsigargin pretreatment (100 nM, 20 min, Ca^2+^-free bath solution) in the absence or presence of Synta66 (5 µM, *n* = 5); *** *p* < 0.0001 (one-way ANOVA with Welch’s correction, Dunnet’s post-hoc test). **C**-**D** Intracellular Ca^2+^ responses to apical (AP, *n* = 5) or basolateral (BL, *n* = 5) application of TRPM7 agonist naltriben (100 µM) to HAT-7 cell monolayers in standard (2 mM Ca^2+^) bath solution; ** *p* < 0.005 (unpaired t-test with Welch’s correction). **E**-**F** Intracellular Ca^2+^ responses to apical (AP, *n* = 7) or basolateral (BL, *n* = 5) application of TRPM7 agonist mibefradil (50 µM) to HAT-7 cell monolayers in standard (2 mM Ca^2+^) bath solution; *** *p* < 0.0001 (unpaired t-test with Welch’s correction). A, C and E show representative single experiments; B, D and F show averaged peak values (mean ± SEM) and statistical significances of differences

Functional polarization of store-operated Ca^2+^ entry (SOCE) in HAT-7 cells grown on Transwell membranes was examined by first depleting the ER stores by pretreatment with thapsigargin (100 nM) in a Ca^2+^-free bath solution. Ca^2+^ was then reintroduced either at the basolateral or apical side. Resulting changes in intracellular Ca^2+^ ([Ca^2+^]_i_) were monitored by fura-2 fluorescence imaging (Fig. 2A and B). Basolateral restoration of extracellular Ca^2+^ resulted in a markedly greater rise in [Ca^2+^]_i_ compared to apical restoration (146.5 ± 11.1% vs. 27.7 ± 4.2% at peak, *n* = 7 and *n* = 5 respectively, *p* < 0.0001). Pretreatment (30 min) and continuous basolateral application of the ORAI1 channel inhibitor Synta66 (5 µM) [8, 15, 16] significantly decreased the peak of the [Ca^2+^]_i_ elevation when tested in the basolateral restoration experiments (146.5 ± 11.1% vs. 50.7 ± 4.0%, *n* = 7 and *n* = 5, respectively; *p* < 0.0001).

TRPM7 activity was examined under the same conditions using two established TRPM7 activators: naltriben [17] and mibefradil [18] (Fig. 2C and D). Naltriben (100 µM) elicited a small but significant increase in [Ca^2+^]_i_ when applied apically, while the response to basolateral application was much smaller (5.7 ± 0.7% vs. 1.3 ± 0.4%, *n* = 5, *p* < 0.005). Similarly, apical activation with 50 µM mibefradil elevated [Ca^2+^]_i_ by 20.2 ± 1% compared to the basolateral response of 2.2 ± 1% (*n* = 7 and *n* = 5 respectively, *p* < 0.0001) (Fig. 2E and F).

### Effects of extracellular and intracellular pH changes on SOCE-mediated Ca^2+^ uptake

**Fig. 3:**
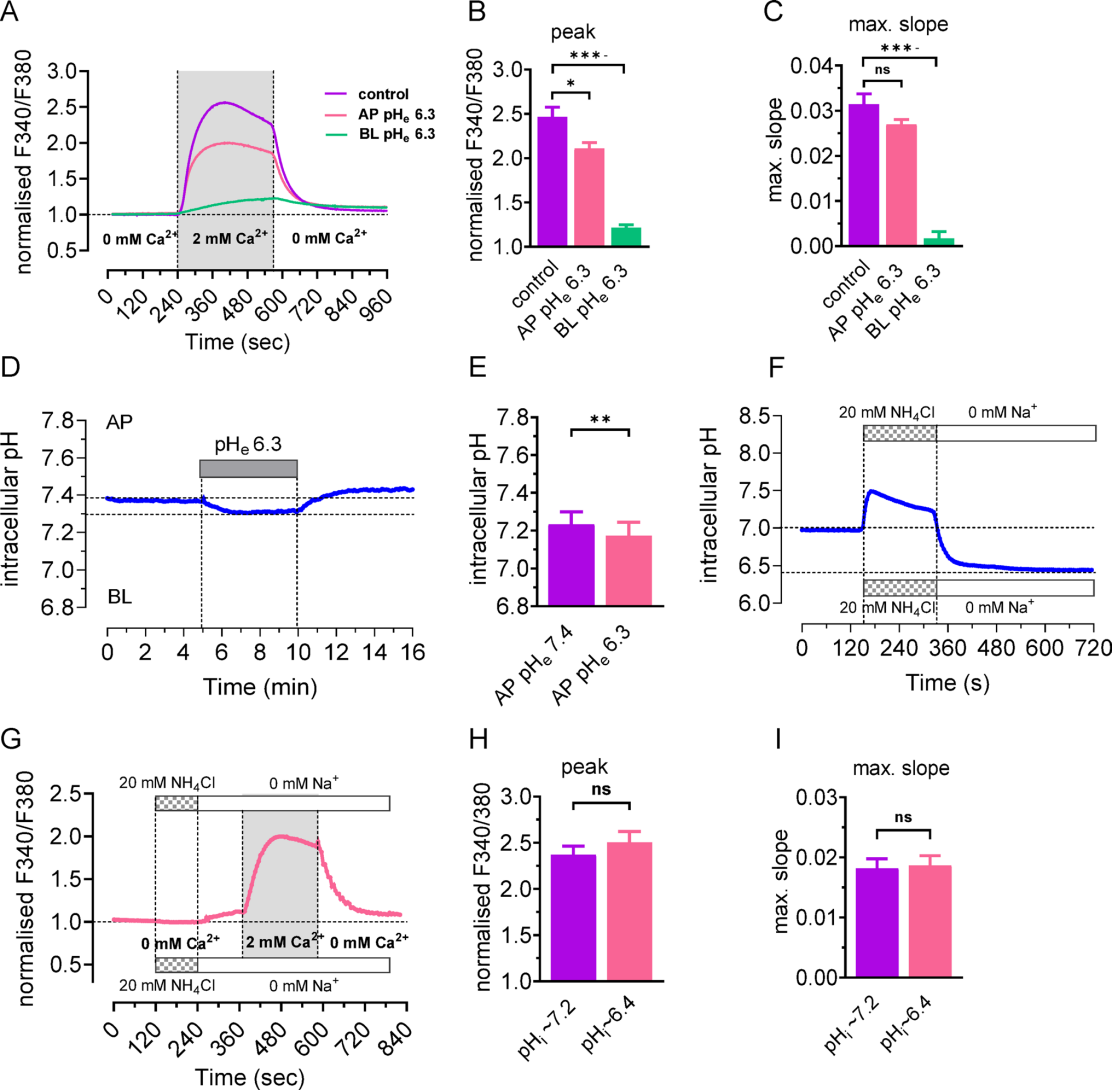
Effects of extra- and intracellular pH changes on intracellular [Ca^2+^] and pH. **A**-**C** [Ca^2+^]_i_ changes elicited by basolateral restoration of extracellular Ca^2+^ in ER store-depleted HAT-7 cells cultured on Transwell membranes. Monolayers were exposed to different apical (AP) and basolateral (BL) extracellular pH values: 7.4 (control, *n* = 7) and 6.3 (*n* = 5). Peak and maximum rising-phase slope values are presented as mean ± SEM; * *p* < 0.05, *** *p* < 0.001 (one-way ANOVA with Welch’s correction, Dunnet’s post-hoc test). **D**-**E** Intracellular pH changes in HAT-7 cell monolayers in response to extracellular acidification (from pH_e_ 7.4 to 6.3) applied to the apical side (paired t-test, ** *p* < 0.01, *n* = 6). **F**-**I** Effects of sustained intracellular acidification (20 mM NH_4_Cl pulse followed by extracellular sodium removal) on pH_i_ (F) and on [Ca^2+^]_i_ following restoration of extracellular Ca^2+^ in ER store-depleted HAT-7 cells (G-I). Peak and maximum slope values are presented as mean ± SEM; ns, no significant difference (unpaired t-test with Welch’s correction, ns; *n* = 5)

Acidification of the solution bathing the apical membrane of ER Ca^2+^ store-depleted HAT-7 cell monolayers, from extracellular pH (pH_e_) 7.4 to 6.3 (Fig. 3A and B), resulted in a significantly reduced peak rise in [Ca^2+^]_i_ following the restoration of extracellular Ca^2+^ at the basolateral membrane: 146.5 ± 11.1% at pH_e_ 7.4 versus 110.7 ± 6.8% at pH_e_ 6.3 (*n* = 7 and *n* = 5 respectively, *p* < 0.05). The initial slope of the [Ca^2+^]_i_ rise, however, was not significantly altered by apical acidification (Fig. 3C). In contrast the application of a similarly acidic bath solution on the basolateral side drastically reduced both the peak (146.5 ± 11.1% at pH_e_ 7.4 vs. 21.6 ± 1.4% at pH_e_ 6.3; *n* = 7 and *n* = 5 respectively, *p* < 0.001) and the slope (0.03136 ± 0.00238 at pH_e_ 7.4 vs. 0.001724 ± 0.000663 at pH_e_ 6.3; *n* = 7 and *n* = 5 respectively, *p* < 0.001) of the [Ca^2+^]_i_ changes (Figs. 3A-C). Collectively, these data suggest that apical acidification has no effect on basolateral store-operated Ca^2+^ entry, as indicated by the unchanged initial slope, but may increase apical Ca^2+^ efflux, resulting in a reduced Ca^2+^ peak.

To assess whether extracellular acidification might modulate Ca^2+^ uptake via a change in intracellular pH (pH_i_), we measured the effect of the same change in pH_e_ on pH_i_ measured using the ratiometric dye BCECF. A reduction in extracellular pH from 7.4 to 6.3, as was applied in the Ca^2+^ uptake experiment shown in Fig. 3A, caused a small but significant acidification of the cytosol by 0.06 ± 0.01 pH units after 5 min (*n* = 6, *p* < 0.01) (Fig. 3D and E).

Next, we used the ammonium pre-pulse technique, a widely used method of intracellular pH manipulation, to investigate the sensitivity of the SOCE-mediated increase in [Ca^2+^]_i_ to a much larger intracellular acidification. Application of NH_4_Cl (20 mM, 2 min) transiently increased pH_i_ in HAT-7 cells and this was followed by a rapid acidification upon withdrawal (Fig. 3F). When followed by substitution of extracellular Na^+^ with impermeant NMDG^+^ to inhibit Na^+^/H^+^ exchange, there was a sustained intracellular acidification at approximately pH_i_ 6.4 [5, 13, 19] while pH_e_ remained unchanged at 7.4.

However, the large and sustained intracellular acidification that followed the NH_4_^+^ pulse (to pH_i_ ~6.4) did not significantly alter either the slope or the peak of the rise in cytosolic Ca^2+^ following the restoration of Ca^2+^ to the basolateral bathing solution (Fig. 3H and I). Unexpectedly, the intracellular acidification at first caused a small but consistent increase in [Ca^2+^]_i_ (Fig. 3G) despite the depletion of intracellular stores and the nominally Ca^2+^-free extracellular environment (4.2 ± 0.6% after 2 min, *n* = 5, one-sample t-test, *p* < 0.01).

## Discussion

Store-operated Ca^2+^ entry and TRPM7 channels have been proposed as major Ca^2+^ uptake pathways in ameloblasts, yet most previous studies were performed in non-polarized cells grown on glass coverslips [4, 5]. Understanding the polarity of Ca^2+^ uptake is essential for elucidating the cellular pathway of vectorial Ca^2+^ transport during enamel maturation. Under non-polarized culture conditions, it is impossible to determine the membrane localization of Ca^2+^ transporters. The present study overcomes this limitation by using HAT-7 cells differentiated on permeable supports, where they form polarized monolayers suitable for independent apical and basolateral perfusion. This approach allows, for the first time, a functional assessment of the Ca^2+^ entry pathways in a polarized ameloblast model.

In our study we show that HAT-7 cells express Orai1–3 and Trpm7 mRNA, and that their expression increases under polarizing conditions. Orai2 was the most abundant of the Orai isoforms, consistent with findings in other epithelial systems and with reports that Orai2 can form functional pore-forming units within SOCE complexes [20, 21]. Interestingly, Orai1 expression was much lower than that of Orai2 and Orai3. This contrasts with earlier observations in rat enamel organ [4], where Orai1 appeared dominant, although Orai2 and Orai3 increased during maturation. A similar predominance of Orai2 and Orai3 over Orai1 was found in LS8, another mouse-derived ameloblast-like cell line [22].

Our functional data reveal that SOCE is strongly polarized to the basolateral membrane, suggesting that the ORAI channels responsible for Ca^2+^ uptake reside predominantly in this membrane domain. This localization aligns well with the physiology of Ca^2+^-secreting epithelia [15, 23], in which Ca^2+^ entry and extrusion occur at the basolateral and apical membranes, respectively. Application of Synta66, a known ORAI1 channel inhibitor, resulted in partial inhibition of basolateral SOCE, suggesting that - despite differences in their mRNA expression levels - multiple ORAI isoforms contribute to basolateral Ca^2+^ entry.

It has also been reported that both secretory- and maturation-stage ameloblasts in Swiss mice express TRPV5 channels, which are known to mediate tubular Ca^2+^ reabsorption in the kidney [24]. However, the predominance of basolateral ORAI-mediated uptake in HAT-7 cells is consistent with observations in another Ca^2+^-secreting epithelium—the mammary gland epithelium—where ORAI channels, rather than TRPV5 (or TRPV6), constitute the principal Ca^2+^ influx pathway [23]. Together, these findings strongly support the idea that ORAI-driven SOCE represents the major basolateral Ca^2+^ uptake mechanism in ameloblasts [2, 25].

During amelogenesis, the continuous and wide fluctuation of the extracellular pH in the enamel matrix is very likely to have an impact on Ca^2+^ transport. In HAT-7 cells, apical acidification from pH_e_ 7.4 to 6.3 significantly reduced the basolateral SOCE-related [Ca^2+^]_i_ peak when extracellular Ca^2+^ was restored following the depletion of intracellular stores. Since intracellular acidification is known to inhibit ORAI channels [26–29], we speculated that lowering extracellular pH at the apical membrane might potentially modulate basolateral SOCE by influencing the pH_i_. Indeed, in our experiments, an extracellular pH drop at the apical side of HAT-7 cells—similar in magnitude to that observed at the apical surface of ameloblasts in vivo—did cause a small, reversible intracellular acidification, which may have inhibited SOCE-mediated Ca^2+^ uptake. However, the smaller [Ca^2+^]_i_ peak may alternatively have been due to extracellular acidification stimulating Ca^2+^ extrusion at the apical membrane rather than inhibiting basolateral SOCE activity. It would, for example, be consistent with enhanced apical PMCA activity, which is known to exchange intracellular Ca^2+^ for extracellular H^+^ and is therefore likely to be accelerated by extracellular acidification.

In attempting to resolve this question we used an ammonium pre-pulse protocol to test the effects of a direct and more robust intracellular acidification on Ca^2+^ entry. Surprisingly, we found that a large and sustained intracellular pH drop did not appear to affect the [Ca^2+^]_i_ peak, suggesting that pH_i_ is not the link between apical pH_e_ changes and basolateral SOCE activity. Again, this interpretation is complicated by the possible effects of the experimental conditions on Ca^2+^ extrusion mechanisms. The substitution of extracellular Na^+^ in this experimental protocol may have inhibited Na^+^-dependent Ca^2+^ extrusion via NCX or NCKX cotransporters [30, 31]. It was also interesting to note that we observed a slight increase in cytosolic [Ca^2+^]_i_ in zero external [Ca^2+^] solutions, prior to the re-addition of Ca^2+^ to evoke SOCE, despite the ER Ca^2+^ stores presumably being depleted. One possible explanation for this is that, during the intracellular acidification, the increase in [H^+^] may displace Ca^2+^ from the binding sites of cytosolic Ca^2+^ buffers, resulting in a small increase in free cytosolic [Ca^2+^]. Thus, the large drop in intracellular pH (ΔpH_i_∼0.8) during the ammonium pre-pulse experiment (Fig. 3F) may have increased free cytosolic Ca^2+^ levels by decreasing Ca^2+^ binding to intracellular proteins [32]. This could explain our observation that a small increase in [Ca^2+^]_i_ occurs in response to intracellular acidification under store-depleted conditions in a Ca^2+^-free environment (Fig. 3G). Thus, the combined effects of decreased Na^+^-dependent Ca^2+^ extrusion and reduced intracellular Ca^2+^ binding could have masked an underlying inhibition of SOCE-mediated Ca^2+^ influx by intracellular acidification in the NH_4_^+^ pulse experiments.

In contrast, basolateral acidification from pH_e_ 7.4 to 6.3 markedly reduced the basolateral SOCE-related [Ca^2+^]_i_ response. These results can be explained by direct inhibition of ORAI channels as previously reported by Malayev et al. [33].

Although it is generally accepted that ORAI channels are blocked by intracellular acidification, recent studies suggest that ORAI homologues have differential sensitivities to changes in intracellular pH [26, 27]. It was previously shown that unlike ORAI1, which is blocked by acidic pH and activated by alkaline pH, the ORAI3/STIM1-activated current is potentiated by protons [34]. More recently, Rychkov et al. has reported that, unlike both ORAI1 and ORAI2, ORAI3 is not sensitive to pH_i_ changes [26].

We have previously demonstrated that functional TRPM7 channels are present in non-polarized HAT-7 cells grown on glass coverslips, suggesting a potential role in Ca^2+^ uptake [5]. To assess the functional polarization of TRPM7, we performed calcium-imaging experiments on HAT-7 cells cultured as a polarized monolayer. Both mibefradil and naltriben, agonists of TRPM7 channels, elicited intracellular Ca^2+^ responses only when applied to the apical side of the monolayer, indicating that these channels are predominantly localized to this membrane region. An important caveat when interpreting the [Ca^2+^]_i_ response is whether these TRPM7-specific drugs have any non-specific effects in these cells. Furthermore, the data in the literature provide no consensus regarding the membrane distribution of TRPM7 proteins. Most immunofluorescence studies report strong cytoplasmic staining without clear apical or basolateral membrane expression, and it is well established that TRPM7 is abundantly localized in intracellular vesicles [35].

Although we have clear evidence from this and our previous study [5] that functional TRPM7 channels are expressed at the apical membrane of HAT-7 cells, the role of TRPM7 in amelogenesis remains incompletely understood. TRPM7 is ubiquitously expressed, and homozygous deletion of its kinase domain results in embryonic lethality [36]. Furthermore, TRPM7 has previously been proposed to facilitate the uptake of the Mg^2+^ and Ca^2+^ required for enamel maturation [6, 7, 37]. However, the predominantly apical TRPM7 activity observed in our experiments suggests that these channels are unlikely to directly mediate transcellular Ca^2+^ transport during amelogenesis in our polarized HAT-7 ameloblast model.

### Limitations

We used a rat-derived ameloblast-like cell line as a model to investigate the transport processes relevant to amelogenesis. Such in vitro systems inherently possess several limitations, including discrepancies between the cellular environments in vitro and in vivo (e.g. differences in cell–cell and cell–matrix interactions), altered gene expression profiles in primary versus immortalized cells, and genetic drift during successive passaging [38], all of which constrain the interpretation and generalizability of our findings.

## Conclusion

We provide here the first functional characterization of polarized Ca^2+^ entry pathways in ameloblast-derived HAT-7 cells grown as confluent, differentiated monolayers on permeable supports. These results demonstrate that store-operated Ca^2+^ entry is mainly localized to the basolateral membrane supporting the concept that this pathway represents the principal route for Ca^2+^ uptake during transepithelial calcium transport in ameloblasts. Gene expression analysis revealed that ORAI2 and ORAI3 transcripts are considerably more abundant than ORAI1 in HAT-7 cells. Nevertheless, pharmacological inhibition with the ORAI1 inhibitor Synta66 significantly reduced SOCE-mediated Ca^2+^ influx. These findings indicate that ORAI1-containing channels make a substantial functional contribution to basolateral Ca^2+^ entry reflecting a key role of ORAI1 within heteromeric ORAI channel complexes or within the functional SOCE machinery. These observations are consistent with the hypothesized role of SOCE in vectorial calcium transport across ameloblast epithelium. In contrast, TRPM7-mediated Ca^2+^ responses were restricted to the apical membrane, suggesting that TRPM7 is unlikely to contribute to Ca^2+^ entry relevant to transepithelial transport. Furthermore, apical acidification had no effect on basolateral SOCE but did reduce the steady-state peak Ca^2+^ response, which would be consistent with enhanced apical Ca^2+^ extrusion. This has important functional implications for vectorial Ca^2+^ transport in ameloblast cells, and thus enamel formation, and warrants further investigation. Extracellular and intracellular pH perturbations may influence intracellular Ca^2+^ dynamics through effects on SOCE, Ca^2+^ extrusion pathways and intracellular buffering. These findings provide an important insight into the molecular mechanism of the transepithelial Ca^2+^ transport pathway involved in enamel formation.

## Supplementary Information

Below is the link to the electronic supplementary material.

Supplementary Material 1

## Data Availability

Data supporting the findings of the study are available on request from the corresponding author.
