# Supplementary material for: Functional model for amelogenesis: polarization and pH sensitivity of calcium uptake in ameloblast-derived HAT-7 cells
Source: Calcif Tissue Int. 2026 Jun 10;117(1):100. doi: 10.1007/s00223-026-01559-x (PMC13249741; doi:10.1007/s00223-026-01559-x)
Supplement: Supplementary file 1 — Supplementary Material 1 [file 223_2026_1559_MOESM1_ESM.docx]

**Functional Model for Amelogenesis: Polarization and pH Sensitivity of Calcium Uptake in Ameloblast-derived HAT-7 Cells**

**Kristóf Kádár^1^, Anna Földes^1^, Róbert Rácz^1^, Susan Van-Weert^1^, Ádám Soós^2^, Jason Bruce^3^, Martin C. Steward^1,3^, Pamela DenBesten^4^, Gábor Varga^1*^ and Ákos Zsembery^1^**

^1^ Department of Oral Biology, Semmelweis University, Budapest, Hungary

^2^ Department of Anatomy, Histology and Embryology, Semmelweis University, Budapest, Hungary

^3^ School of Medical Sciences, Faculty of Biology, Medicine & Health, University of Manchester, Manchester, UK

^4^ Department of Orofacial Science, University of California, San Francisco, USA

**^*^Corresponding Author**

Dr. Gábor Varga

Postal address: H-1089 Budapest, Nagyvárad tér 4, Hungary

Tel.: +36-20 825-0604

E-mail address: varga.gabor@semmelweis.hu

**Supplementary information:**


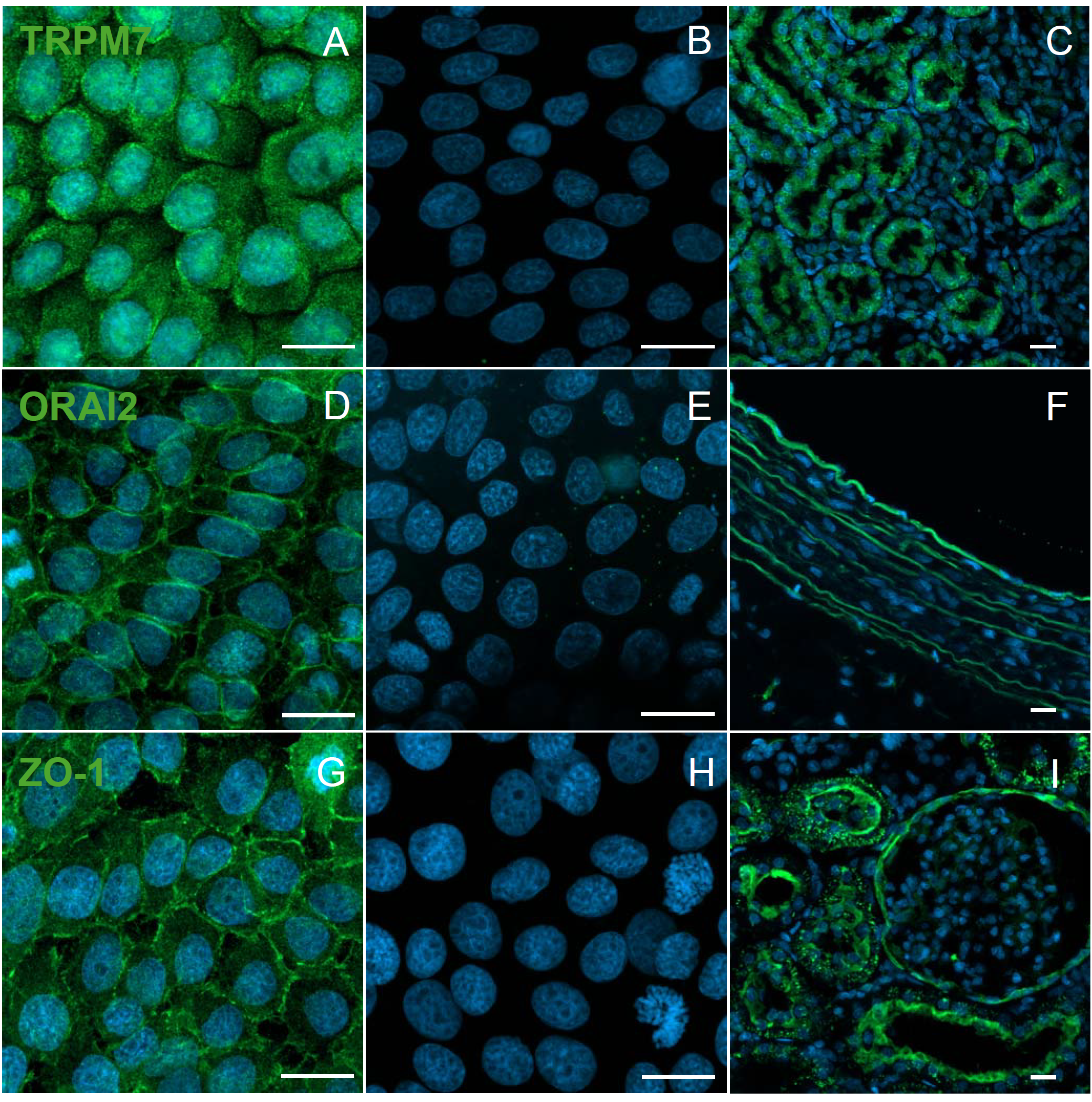


***Fig. S1*** Immunolocalization of TRPM7 (A), ORAI2 (D), and ZO-1 (G) proteins (green) in polarized HAT-7 cells cultured on Transwell membranes under differentiating conditions. Corresponding negative control stainings are shown in panels B, E, and H. Positive control stainings are shown in rat kidney cryosections for TRPM7 [1] and ZO-1 [2,3] (C, I) and in rat aortic wall tissue for ORAI2 [4] (F). Nuclei were counterstained with DAPI (blue). Scale bar: 20 μm in all panels.

**Immunohistochemistry**

For positive control stainings, cryosections (12 μm) were prepared from 4% paraformaldehyde-fixed rat tissues. Microwave heating was applied, followed by permeabilization with 3 % Triton X-100 for 15min. Slides were incubated overnight at 4°C with rabbit anti-ZO-1 (Invitrogen, 40-2200, 6 μg/ml) or monoclonal ORAI2 (G-5) antibody (Santa Cruz, sc-376757, 1:50) or with rabbit anti-TRPM7 (Thermo Fisher Scientific, ACC-047; 1:50). Alexa Fluor 488 anti-rabbit secondary antibodies (ZO-1, TRPM7) or Alexa Fluor 488 anti-mouse secondary antibodies (ORAI2) were applied for 1 h at RT, respectively. Nuclei were counterstained with DAPI (1 µg/ml working concentration; Merck) for 15 min. TrueBlack^®^ Lipofuscin Autofluorescence Quencher (DMSO stock, Cat. No. 23011) diluted 1:30 in 70% EtOH was applied for 2 min at RT to reduce autofluorescence. Sections were imaged by confocal laser scanning microscopy (Zeiss LSM 900) and stimulated emission depletion (STED, Abberior Expert Line - Nikon Ti2) microscopy using CellSens (Evident/Olympus) and ZEN Imaging (Zeiss) software.

**References**

1. Yee NS, Kazi AA, Yee RK (2014) Cellular and developmental biology of TRPM7 channel-kinase: Implicated roles in cancer. Cells (Basel, Switzerland) 3:751-777.

2. Qiao X, Roth I, F+ęraillAe E, Hasler U (2014) Different effects of ZO-1, ZO-2 and ZO-3 silencing on kidney collecting duct principal cell proliferation and adhesion. Cell Cycle (Georgetown, Tex.) 13:3059-3075.

3. Rincon-Choles H, Vasylyeva TL, Pergola PE, Bhandari B, Bhandari K, Zhang JH, Wang W, Gorin Y, Barnes JL, Abboud HE (2006) ZO-1 expression and phosphorylation in diabetic nephropathy. Diabetes 55:894-900.

4. Wei Y, Bai S, Yao Y, Hou W, Zhu J, Fang H, Du Y, He W, Shen B, Du J (2021) Orai-vascular endothelial-cadherin signaling complex regulates high-glucose exposure-induced increased permeability of mouse aortic endothelial cells. BMJ Open Diabetes Research & Care 9:e002085
